# Insights into the Mechanisms of Protective Immunity against *Cryptococcus neoformans* Infection Using a Mouse Model of Pulmonary Cryptococcosis

**DOI:** 10.1371/journal.pone.0006854

**Published:** 2009-09-03

**Authors:** Karen L. Wozniak, Sailatha Ravi, Sandra Macias, Mattie L. Young, Michal A. Olszewski, Chad Steele, Floyd L. Wormley

**Affiliations:** 1 Department of Biology, The University of Texas at San Antonio, San Antonio, Texas, United States of America; 2 The South Texas Center for Emerging Infectious Diseases, The University of Texas at San Antonio, San Antonio, Texas, United States of America; 3 VA Ann Arbor Health System, University of Michigan Health System, Ann Arbor, Michigan, United States of America; 4 Division of Pulmonary & Critical Care Medicine, Department of Internal Medicine, University of Michigan Health System, Ann Arbor, Michigan, United States of America; 5 Department of Medicine, University of Alabama at Birmingham, Birmingham, Alabama, United States of America; Research Institute for Children and the Louisiana State University Health Sciences Center, United States of America

## Abstract

*Cryptococcus neoformans* is an opportunistic fungal pathogen that causes life-threatening pneumonia and meningoencephalitis in immune compromised individuals. Previous studies have shown that immunization of BALB/c mice with an IFN-γ-producing *C. neoformans* strain, H99γ, results in complete protection against a second pulmonary challenge with an otherwise lethal cryptococcal strain. The current study evaluated local anamnestic cell-mediated immune responses against pulmonary cryptococcosis in mice immunized with *C. neoformans* strain H99γ compared to mice immunized with heat-killed *C. neoformans* (HK*C.n.*). Mice immunized with *C. neoforman*s strain H99γ had significantly reduced pulmonary fungal burden post-secondary challenge compared to mice immunized with HK*C.n.* Protection against pulmonary cryptococcosis was associated with increased pulmonary granulomatous formation and leukocyte infiltration followed by a rapid resolution of pulmonary inflammation, which protected the lungs from severe allergic bronchopulmonary mycosis (ABPM)-pathology that developed in the lungs of mice immunized with HK*C.n*. Pulmonary challenge of interleukin (IL)-4 receptor, IL-12p40, IL-12p35, IFN-γ, T cell and B cell deficient mice with *C. neoformans* strain H99γ demonstrated a requirement for Th1-type T cell-mediated immunity, but not B cell-mediated immunity, for the induction of H99γ-mediated protective immune responses against pulmonary *C. neoformans* infection. CD4^+^ T cells, CD11c^+^ cells, and Gr-1^+^ cells were increased in both proportion and absolute number in protected mice. In addition, significantly increased production of Th1-type/pro-inflammatory cytokines and chemokines, and conversely, reduced Th2-type cytokine production was observed in the lungs of protected mice. Interestingly, protection was not associated with increased production of cytokines IFN-γ or TNF-α in lungs of protected mice. In conclusion, immunization with *C. neoformans* strain H99γ results in the development of protective anti-cryptococcal immune responses that may be measured and subsequently used in the development of immune-based therapies to combat pulmonary cryptococcosis.

## Introduction

*Cryptococcus neoformans*, the etiological agent of cryptococcosis, is an opportunistic fungal pathogen that typically affects individuals with impaired T cell function (i.e., individuals with AIDS, lymphoid malignancies, and recipients of immunosuppressive therapies) [1]–[5]. Clinical and experimental studies have suggested that protection against cryptococcosis is mediated by T helper (Th) 1–type CD4^+^ T cell-mediated immunity (CMI) [3], [5]–[13]. Protection against cryptococcosis is induced following the production of Th1-type cytokines including IL-12 and IFN-γ, as well as the pro-inflammatory cytokine TNF-α [14]–[23]. Neutralization of IFN-γ, IL-12, and/or TNF-α in mice results in increased susceptibility to cryptococcal infection [14]–[16], [21]. On the other hand, production of Th2-type cytokines, such as IL-4, IL-5, IL-10, and IL-13, are associated with exacerbation of disease in animal models [15], [23]–[25].

To date, there is no vaccine or immunotherapy approved for the prevention of cryptococcosis. Vaccination of mice with various protein preparations has been shown to induce partial protection and delayed-type hypersensitivity (DTH) responses against subsequent challenge [26]–[31]. Experimental studies in mice have also shown that passive administration of anti-cryptococcal mAbs prolongs survival and reduces fungal burden compared to control animals [32], [33]. In addition, recombinant Th1-type cytokines have been investigated for their potential as adjunctive antifungal chemotherapy and to enhance anti-cryptococcal host immune responses [18], [34]–[37]. Specifically, studies with IFN-γ have yielded some promising results as both clinical and experimental studies show that adjunctive therapy in combination with antifungal agents enhances clearance of the organism [17], [36], [38], [39]. Unfortunately, these studies have been unable to demonstrate complete protection against subsequent *C. neoformans* challenge.

Recent studies in our laboratory have shown that an acute infection with a *C. neoformans* strain H99 engineered to express IFN-γ, designated *C. neoformans* strain H99γ, results in higher Th1-type cytokine and chemokine expression, lower pulmonary fungal burden, and increased pulmonary leukocyte recruitment compared to mice infected with wild-type *C. neoformans* [40]. Moreover, prior immunization with C. *neoformans* strain H99γ, but not heat-killed *C. neoformans* (HK*C.n.*), results in the induction of sterilizing immunity against a subsequent lethal pulmonary challenge with wild-type cryptococci in mice [40], [41]. Although *C. neoformans* strain H99γ is able to induce protection in the mouse model of cryptococccal infection, it has no attenuation in growth and expresses the full complement of cryptococcal virulence factors [40]. The purpose of the current study was to use this model system to determine the immune parameters associated with protection in mice immunized with *C. neoformans* strain H99γ and given a secondary pulmonary challenge with wild-type *C. neoformans*.

## Results

### Protection against secondary challenge is afforded by immunization with *C. neoformans* strain H99γ

**Figure 1:**
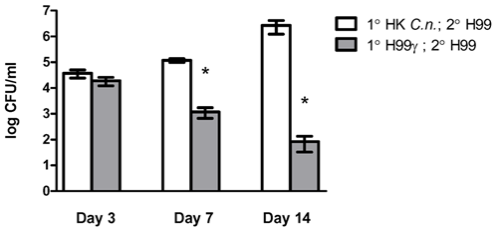
Protection against experimental pulmonary cryptococcosis following secondary challenge. BALB/c mice were immunized with either heat-killed *C. neoformans* (HK*C.n.*) (white bars) or *C. neoformans* strain H99γ (gray bars), allowed 100 days to resolve the infection, and subsequently given a second challenge with *C. neoformans* strain H99. Lungs were excised at days 3, 7, and 14 post-secondary inoculation, and the cryptococcal burden was quantified. Results are expressed as mean log CFU per milliliter ± standard errors of the means. Asterisks (*) indicate where significant decreases (*P*<0.01) in CFU were observed compared to mice immunized with HK*C.n.* Pulmonary fungal burden data are cumulative of three experiments using 5 mice per time point. Separate mice were used for each time point.

Our previous studies demonstrated that prior challenge of BALB/c mice with an IFN-γ producing *C. neoformans* strain, H99γ, but not HK*C.n.*, results in the development of full protection against a second otherwise lethal challenge with the fully virulent *C. neoformans* strain H99 [40], [41]. To further characterize the development of anamnestic immune responses following these immunization regimens, BALB/c mice were inoculated with either *C. neoformans* strain H99γ or HK*C.n.* intranasally and allowed 100 days to mount an immune response and to resolve the infection. Both groups of animals were subsequently challenged with wild-type *C. neoformans* strain H99 that does not produce IFN-γ, and the fungal burden was quantified on days 3, 7, and 14 post-inoculation. Pulmonary infection using the same or a 10-fold lower inoculum of wild-type *C. neoformans* strain H99 in mice results in fatal infection (unpublished observations) thereby necessitating that we use HK*C.n.* for the vaccination control as in previous studies [40], [41]. Mice immunized with HK*C.n.* showed progressive growth of *C. neoformans* strain H99 in the lungs (Figure 1). In contrast, mice immunized with *C. neoformans* strain H99γ showed significantly reduced pulmonary fungal burden on days 7 and 14 post-secondary inoculation (p<0.0001) compared to mice immunized with HK*C.n.* (Figure 1). These data corroborate our previous reports and demonstrate the reproducibility of this model [40], [41]. For clarity, mice immunized with *C. neoforman*s strain H99γ or HK*C.n*. yeast will hereafter be referred to as protected and non-protected mice, respectively.

### Mice immunized with *C. neoformans* strain H99γ but not HK*C.n.* are protected from allergic bronchopulmonary mycosis (ABPM) pathology during secondary *C. neoformans* infection

**Figure 2:**
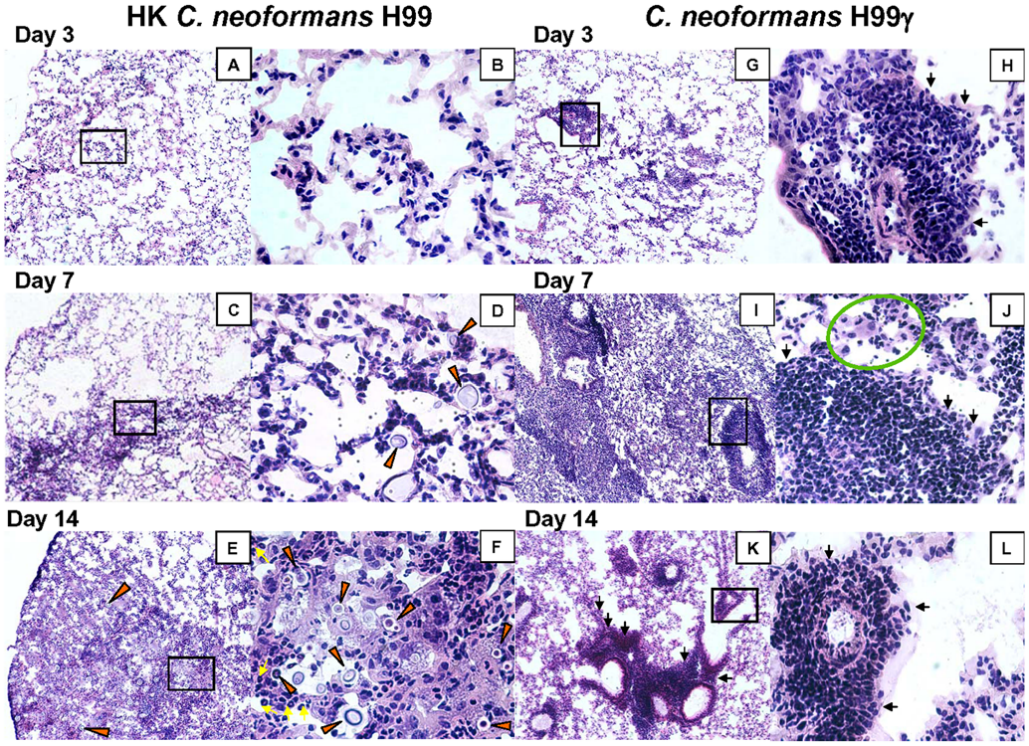
Progression of pathological changes in the lungs of HK*C.n.-* and *C. neoformans* strain H99γ-immunized mice following secondary challenge with wild-type cryptococci. Histological samples were prepared as described in Methods. Digital photographs of H&E stained slides were taken under the light microscope at 10X and 40X power objectives. Note the progressive growth and spread of *C. neoformans* (red arrows), concurrent development of “loose” inflammatory infiltrates (A–F) in the lungs of non-protected mice, and the hallmarks of allergic broncho-pulmonary mycosis (ABPM) pathology [eosinophilia (yellow arrows) and extended macrophages harboring live organisms (E–F)]. In protected mice, note rapid formation of “tight” mononuclear infiltrates and the absence of *C. neoformans* growth in the alveolar space (G–J) and a rapid resolution of the inflammatory response that subsides to the well marginalized “cuffs” of neolymphatic tissue within the bronchovascular bundle (black arrows) with the concurrent clearance of inflammation from the alveolar space (K, L). Data are representative of 4 experiments using 3 mice per experiment.

Our data show significant differences in microbial clearance between non-protected versus protected mice following challenge with *C. neoformans* strain H99. Our next goal was to compare the effects of these immunization regimens on: 1) the development of inflammatory infiltrates in different micro-anatomical lung compartments; and 2) the development of lung pathology during secondary infection with wild-type cryptococci. Lung sections were collected on days 3, 7, and 14 post-secondary infection with *C. neoformans* strain H99, stained with H&E, and analyzed by light microscopy. Comparison of the histological samples demonstrated that the development of the inflammatory response in non-protected mice (Fig. 2A–F) was delayed in comparison with that of protected mice (Fig. 2G–L), consistent with the cytology data. However, the proportion of lungs involved in the inflammatory response continued to increase throughout the analyzed time course in non-protected mice (Fig. 2A, C, E). In contrast, the extent of the inflammatory response peaked on day 7 in the protected mice (Fig. 2G, I, K), with the considerable resolution on day 14.

We next analyzed the micro-anatomical distribution and character of these inflammatory infiltrates in both groups of mice. Leukocyte recruitment in the non-protected mice was diffuse and initially concentrated in the alveolar septa/parenchyma, while growing *C. neoformans* organisms remained unaccompanied by the inflammatory cells within the alveolar air space (Fig. 2D). In contrast, protected mice demonstrated rapid recruitment of cells into the bronchovascular bundles and rapid formation of tight mononuclear infiltrates (Fig. 2H&L ), consistent with mixed lymphocyte/monocytes/DC infiltrates observed during protective Th1/DTH responses in *C. neoformans* infected lungs [42], [43]. By day 7 post- challenge, cryptococci were deeply buried within regions of granulomatous inflammation, and are virtually unrecognizable. At this time numerous macrophages are present within the alveoli, many showing small ingested particles, most likely, the destroyed cryptococci (Fig. 2J, green circle).

In terms of the development of lung pathology, at day 14 post-challenge we also observed major differences between non-protected versus protected mice. Lungs from non-protected mice showed a large area of diffuse inflammation with widespread cryptococcal growth (Fig. 2E). The cellular infiltrates were composed of eosinophils, and large/extended macrophages, of which many harbor cryptococcal organisms with large capsules and budding yeasts (Fig. 2F). These pathologies are consistent with ABPM-type pathologies described during Th2-driven responses in *C. neoformans* infected lungs [44], [45].

In contrast with the severe pathology observed in non-protected animals, the protected mice showed rapid resolution of inflammation from the alveolar space (Fig. 2K). The mononuclear infiltrates were still present in bronchovascular bundles, but these infiltrates showed clear demarcation from the majority of the alveolar area, in which few *C. neoformans* organisms and inflammatory cells were present (Fig. 2K–L). The lungs of protected mice showing resolving inflammation demonstrates that immunization with *C. neoformans* strain H99γ protected lungs from the severe ABPM-pathology that developed in the lungs of HK*C.n.*-immunized animals challenged with wild-type cryptococci.

### Immunization with *C. neoformans* H99γ results in rapid pulmonary recruitment of leukocytes during secondary response to challenge with wild-type *C. neoformans*

We next compared pulmonary leukocyte recruitment in non-protected versus protected mice on days 3, 7, and 14 post-secondary inoculation with *C. neoformans* strain H99. We quantified numbers of CD45^+^ leukocytes, MHC class II^+^ antigen-presenting cells (APCs), Gr-1^+^ cells, F4/80^+^ cells, and CD11c^+^ cells in enzymatically dispersed lungs at these time points. Our specific gating strategy for Gr-1^+^ cells concentrated on cells that expressed high levels of Gr-1 and were also CD11b^+^ and CD11c^-^, which are suggestive of neutrophils [46]–[49] (data not shown). We also gated specifically on cells expressing high levels of F4/80^+^ that were also CD11b^+^, Gr-1^−^, and CD11c^−^ which are suggestive of macrophages [46], [49] (data not shown).

**Figure 3:**
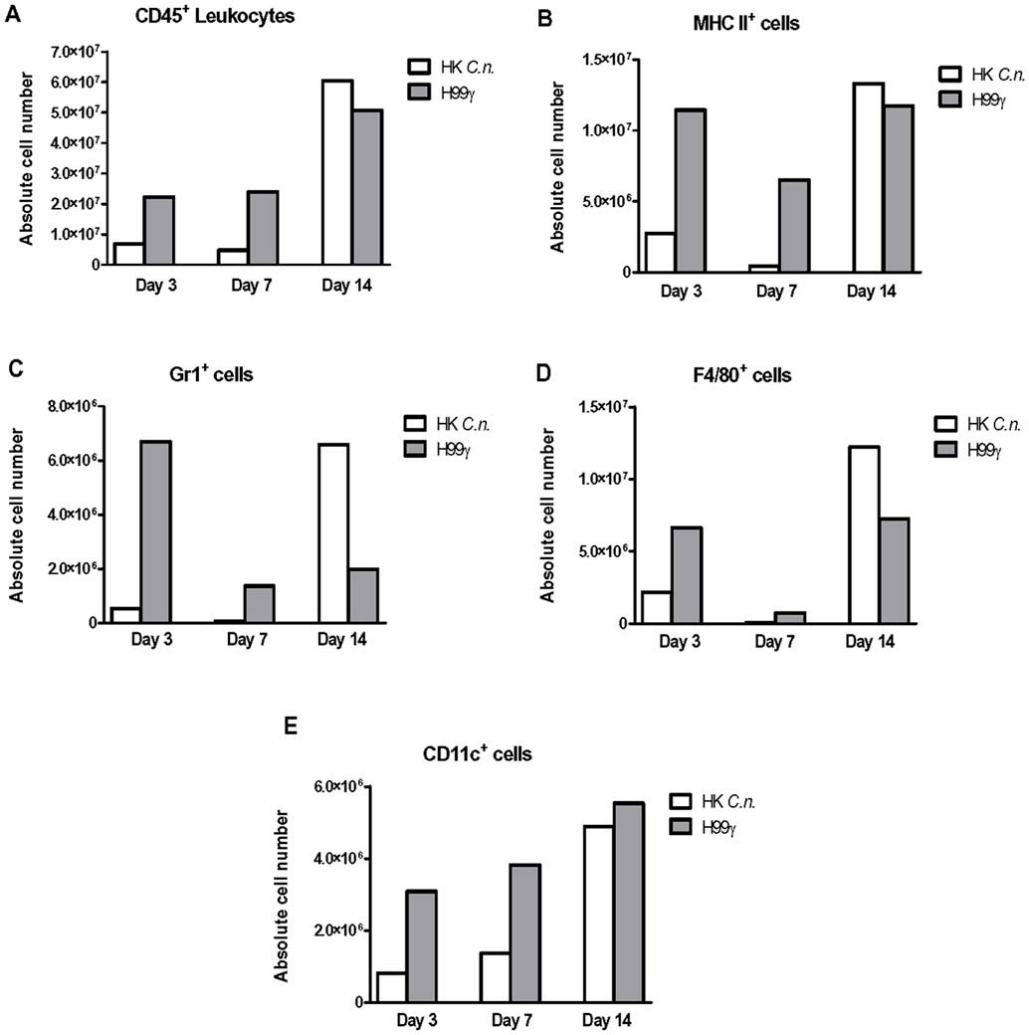
Pulmonary inflammatory leukocyte populations following secondary challenge. BALB/c mice received an immunization with either HK*C.n.* (white bars) or *C. neoformans* strain H99γ (gray bars), allowed 100 days to resolve the infection, and subsequently given a second challenge with *C. neoformans* strain H99. Leukocytes were labeled with anti-CD45 antibodies (A) or dual labeled with anti-IA/IE (MHC class II) and anti-CD45 (B), anti-Gr-1 and anti-CD45 (C), anti-F4/80 and anti-CD45 (D), or anti-CD11c (E) and analyzed by flow cytometry. Data shown are absolute cell numbers from one representative experiment of 3 experiments performed using 5 mice per group per experiment.

The percentages of Gr-1^+^ and CD11c^+^ cells were significantly increased at day 3 post-secondary challenge in protected mice compared to non-protected mice (Table 1). Concurrent with clearance of the organism, the percentage of CD11c^+^ cells and MHC class II^+^ APCs was significantly decreased in protected mice at day 14 post-secondary challenge compared to non-protected mice. However, when we examined the absolute total number of pulmonary leukocytes (shown as representative data in Figure 3), we found increased CD45^+^ cells at days 3 and 7 post-secondary challenge in protected mice compared to non-protected mice (Figure 3A). In addition, we observed an increase in the total number of MHC class II^+^ APCs (Figure 3B) in protected mice on days 3 and 7 post-secondary inoculation compared to non-protected mice. Analysis of phagocyte subsets during the same time course showed a greater absolute number of Gr-1^+^ cells and CD11c^+^ cells in lung tissues of protected mice compared to non-protected mice on days 3 and 7 post-secondary inoculation (Figure 3C and 3E). The absolute number of F4/80^+^ cells was increased on day 3 post-secondary challenge in protected mice compared to non-protected mice (Figure 3D). These results strongly suggest that immunization of BALB/c mice with *C. neoformans* strain H99γ results in the early recruitment of inflammatory leukocytes and APCs into the lungs in response to a subsequent pulmonary cryptococcal challenge.

**Table 1:** Percent pulmonary cell populations following secondary challenge.

Cell type	HK*C.n.*	H99γ	HK*C.n.*	H99γ	HK*C.n.*	H99γ
	Day 3	Day 3	Day 7	Day 7	Day 14	Day 14
F4/80^+^	9.2±1.5	8.9±1.2	3.5±1.5	2.6±0.6	4.8±0.9	3.9±0.6
CD11c^+^	7.3±1.0	11.2±1.7*	7.5±1.9	13.8±4.1	13.9±1.5	8.5±1.0*
MHC II^+^	23.3±1.9	39.2±10.1	28.0±3.5	33.9±2.2	40.0±1.5	25.6±3.8*
Gr1^+^	2.8±0.6	10.8±1.8*	3.2±1.4	4.1±0.8	5.5±0.6	5.4±2.2
CD4^+^	15.9±3.1	19.9±3.8	17.5±2.8	41.2±3.7*	25.7±1.5	34.9±5.1
CD8^+^	4.1±1.0	4.4±1.1	5.3±1.0	5.2±0.9	4.0±1.0	3.7±0.8

* =  significant differences HK*C.n.* vs. H99γ (p<0.05).

### Protection afforded by immunization with *C. neoformans* H99γ requires T lymphocytes and Th1-type cytokine production

**Figure 4:**
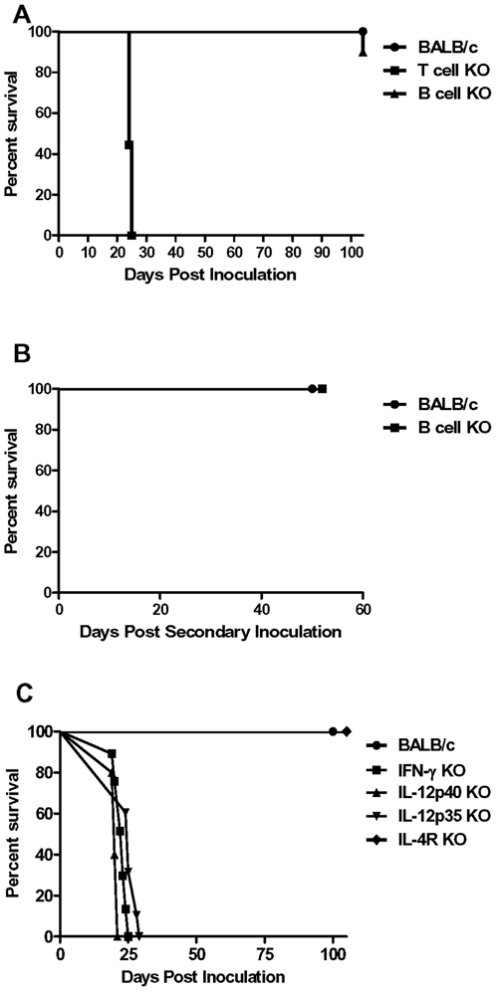
Pathogenesis of *C. neoformans* strain H99γ in T cell and B cell deficient mice. A. Wild-type (circles), T cell deficient (squares), and B cell deficient (triangles) mice were given an intranasal inoculation with *C. neoformans* strain H99γ. Survival data shown are from one experiment using 10 mice per group. B. Wild-type (circles) and B cell deficient (squares) mice that survived an initial inoculation with *C. neoformans* strain H99γ received a second challenge with the parental *C. neoformans* strain H99. Survival data shown are from one experiment using 9–10 mice per experimental group. C. Wild-type (circles), IFN-γ deficient (squares), IL-12p40 deficient (upward triangles), IL-12p35 deficient (downward triangles), and IL-4R deficient (diamonds) mice were given an intranasal inoculation with *C. neoformans* strain H99γ. Survival data shown are from one experiment each, using 10–15 mice per group.

Our next goal was to determine if B and/or T cells were required to generate protective anti-cryptococcal immunity in response to pulmonary H99γ infection. We evaluated survival of B- and T-cell deficient mice infected with *C. neoformans* strain H99γ. The results indicated 100% and 90% survival of wild-type mice and B-cell deficient mice, respectively. This contrasted with 100% mortality observed in *C. neoformans* strain H99γ-infected T-cell deficient mice which had a median survival time of 24 days (Figure 4A, *P*<0.0001 compared to wild-type and B-cell deficient mice). Furthermore, the recovered B-cell deficient and wild-type mice were completely protected against the secondary challenge with wild-type *C. neoformans* strain H99 (Figure 4B). Cultures of lung and brain tissues of the re-challenged mice did not have detectable viable cryptococci at the termination of the survival experiment (day 60).

Previous studies demonstrated that pulmonary infection of mice with *C. neoformans* strain H99γ results in the stimulation of local Th1-type anti-crytococcal CMI responses [40]. We thus aimed to determine if protection generated in mice against pulmonary *C. neoformans* strain H99γ infection was indeed due to the induction of Th1-type cytokine production. Consequently, the virulence of *C. neoformans* strain H99γ was assessed in immune competent BALB/c mice compared to immune compromised mice, including IL-4 receptor(R) deficient mice that are unable to mount optimal Th2-type cytokine responses as well as IL-12p40, IL-12p35, and IFN-γ deficient mice that are unable to mount Th1-type DTH responses. The results indicated 100% survival of BALB/c mice and IL-4R deficient mice (Figure 4C). Cultures of lung and brain tissues of the surviving mice were negative for viable *C. neoformans* at the completion of the survival experiment (day 100). In contrast, IL-12p40, IL-12p35, and IFN-γ deficient mice each experienced 100% mortality following infection with *C. neoformans* strain H99γ. Median survival times were 20, 25, and 23 days post *C. neoformans* strain H99γ infection for IL-12p40, IL-12p35, and IFN-γ deficient mice, respectively (Figure 4C; *P*<0.0001 compared to immune competent BALB/c and IL-4R deficient mice). Thus, the induction of protective immunity following pulmonary immunization with *C. neoformans* strain H99γ appears to require the induction of Th1-type cell-mediated immune responses.

### Pulmonary lymphocyte recruitment during pulmonary cryptococcosis in immunized mice

**Figure 5:**
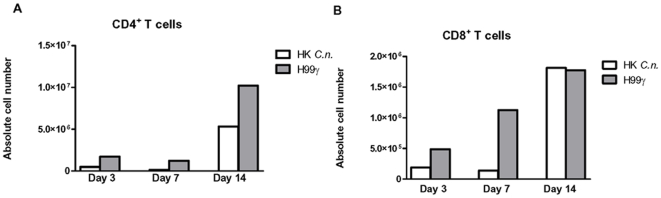
Pulmonary T lymphocyte populations following secondary challenge. BALB/c mice were immunized with either HK*C.n.* (white bars) or *C. neoformans* strain H99γ (gray bars) and were allowed 100 days to resolve the infection. Subsequently, mice were given a second challenge with *C. neoformans* strain H99. Pulmonary leukocytes were dual-labeled with anti-CD4 and anti-CD3 antibodies (A), or anti-CD8 and anti-CD3 antibodies (B) and analyzed by flow cytometry. Data shown are absolute cell numbers from one representative experiment of 3 experiments performed using 5 mice per group per experiment.

To determine the capacity of *C. neoformans* H99γ-immunized mice (protected mice) to stimulate lymphocyte infiltration following a second experimental pulmonary challenge compared to non-protected mice, total leukocytes were isolated from lung tissues on days 3, 7, and 14 post secondary challenge with wild-type *C. neoformans*, and the lymphocyte subpopulations characterized by flow cytometry. Results examining percent cell infiltration showed significantly increased CD4^+^ T cells in protected mice at day 7 post-challenge compared to non-protected mice (Table 1). When absolute cell numbers were examined (shown as representative data in Figure 5), protected mice had a higher absolute number of CD4^+^ T lymphocytes (Figure 5A) in lung tissues on days 3, 7, and 14 post-secondary challenge compared to non-protected mice. CD8^+^ T lymphocytes (Figure 5B) were present in higher numbers in lung tissues on days 3 and 7 post-secondary challenge in protected mice compared to non-protected mice.

### Analysis of lymphocyte populations within pulmonary granulomatous regions during *C. neoformans* infection

**Figure 6 pone-0006854-g006:**
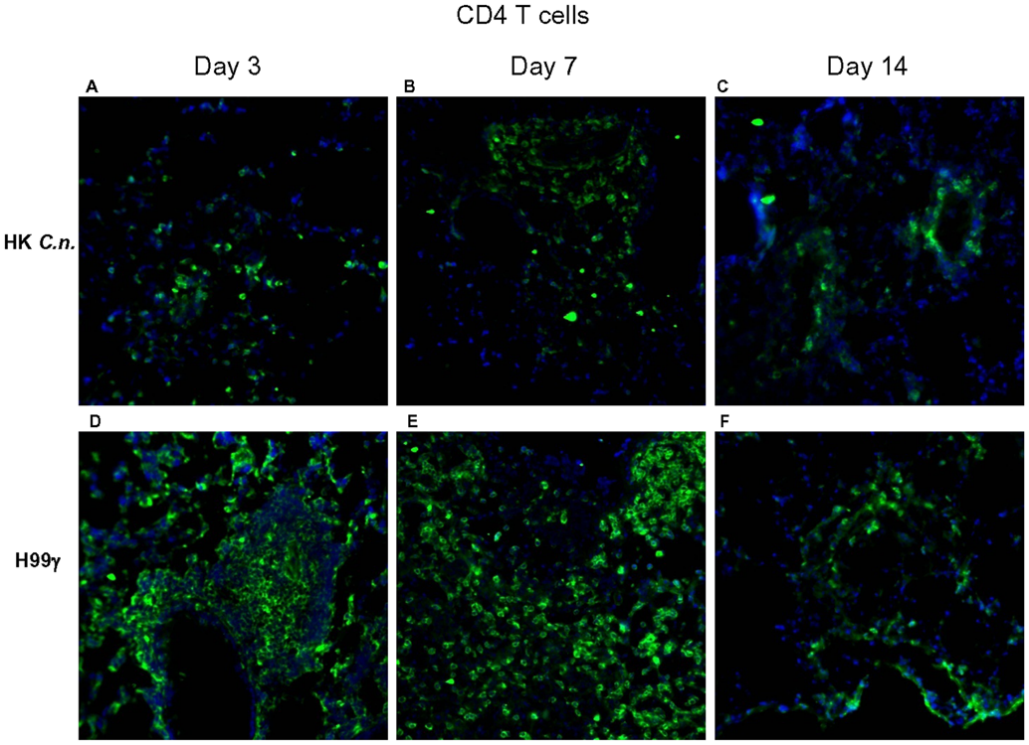
Immunohistochemistry of CD4^+^ T cells in granulomatous regions. BALB/c mice were immunized with either HK*C.n.* or *C. neoformans* strain H99γ, allowed to resolve the infection, and subsequently given a second challenge with *C. neoformans* strain H99. Lung sections frozen in OCT medium were subsequently cryosectioned and evaluated for immunoflourescent staining using anti-CD4 antibodies (green). Sections were counterstained using DAPI (blue). Protected mice show increased CD4^+^ cell staining in granulomatous regions at days 3 (D) and 7 (E) post-secondary challenge compared to non-protected mice (A) and (B). Data shown are representative lung sections from 4 independent experiments using 3 mice per experiment. Magnification  =  original×20.

**Figure 7:**
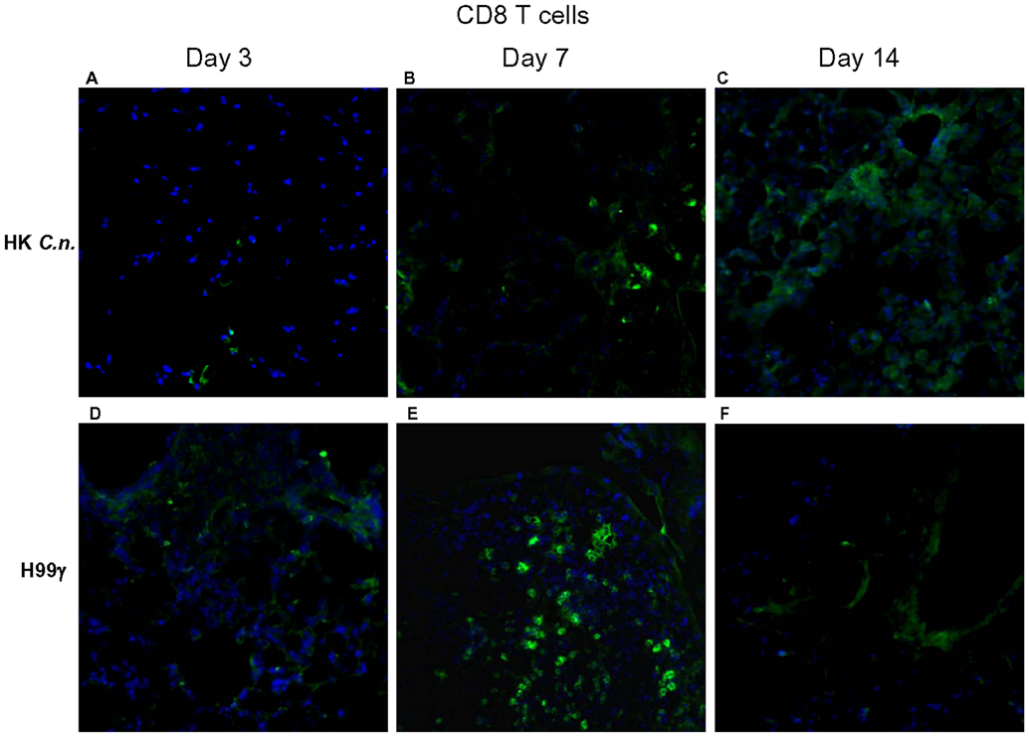
Immunohistochemistry of CD8^+^ T cells in granulomatous regions. BALB/c mice were immunized with either HK*C.n.* or *C. neoformans* strain H99γ, allowed to resolve the infection, and subsequently given a second challenge with *C. neoformans* strain H99. Lung sections frozen in OCT medium were subsequently cryosectioned and analyzed for immunoflourescent staining using anti-CD8 antibodies (green). Sections were counterstained using DAPI (blue). Protected mice show increased CD8^+^ cell staining in granulomatous regions at day 7 (E) post-secondary inoculation compared to non-proteced mice (B). Data shown are representative lung sections from 4 independent experiments using 3 mice per experiment. Magnification  =  original×20.

Pulmonary T cell populations within specific granulomatous regions of whole lungs derived from protected and non-protected mice at days 3, 7, and 14 post-secondary inoculation were evaluated using immunofluorescence staining. An increase in CD4^+^ T cells within granulomatous regions of protected mice was observed on days 3 and 7 post-secondary inoculation (Figures 6D and E) compared to non-protected mice (Figures 6A and B). Protected mice also appeared to have increased CD8^+^ T cells in lung granulomatous regions at day 7 post-secondary challenge (Figure 7E) compared to non-protected mice (Figure 7B). The reduction of specific lymphocyte subpopulations (CD4^+^ T cells and CD8^+^ T cells) within pulmonary granulomatous regions of protected mice on day 14 post-secondary inoculation correlated with a reduction in fungal burden (Figure 1) and total lung granulomatous regions (Figure 2K).

### Pulmonary cytokine expression during experimental cryptococcosis in lungs of protected mice

To evaluate local cytokine responses, lung homogenates were prepared from pulmonary tissues excised from protected and non-protected mice on days 3, 7, and 14 post-secondary inoculation and evaluated for Th1-type (IL-2, IL-12p70, IFN-γ), Th2-type (IL-4, and IL-5), pro-inflammatory cytokine production (IL-1α, IL-1β, IL-17, TNF-α, and G-CSF) as well as chemokine production (MIP-1α, MIP-1β, MCP-1, and RANTES). As shown in Table 2, Th1-type (IL-2 and IL-12p70) and pro-inflammatory (IL-1α, IL-1β, IL-17, and G-CSF) cytokine and chemokine (MIP-1α, MIP-1β, MCP-1, KC, and RANTES) levels were significantly higher and, conversely, Th2-type (IL-4, IL-5, and IL-10) cytokine levels were significantly lower in lung homogenates derived from protected mice compared to non-protected mice during a secondary pulmonary *C. neoformans* infection. The increases in Th1-type cytokine and chemokine expression in protected mice closely mirrored the increases in pulmonary leukocyte infiltration (Figures 2, 3, 5–7) and subsequent reductions in pulmonary fungal burden (Figure 1) observed during secondary challenge. Additionally, the protected mice showed no signs of an exacerbated Th1-type cytokine response at day 14 post-challenge, concurrent with pulmonary organism clearance (Figure 1). Interestingly, the production of IFN-γ and TNF-α, two cytokines previously shown to be critical for the induction of protection against acute pulmonary *C. neoformans* infection, were not significantly increased in protected compared to non-protected mice.

**Table 2:** Pulmonary Th1/Th2, inflammatory cytokine and chemokine levels^a^.

Cytokine	HK*C.n.*	H99γ	HK*C.n.*	H99γ	HK*C.n.*	H99γ
	Day 3	Day 3	Day 7	Day 7	Day 14	Day 14
**Th1-type**						
IL-2	6.0±1.3	74.0±18.4*	17.6±1.7	29.7±3.1*	9.4±0.9	5.8±0.5*
IL-12p70	11.8±3.5	56.0±13.0*	20.9±1.5	104.8±18.3*	51.4±9.8	11.4±1.0*
IFN-γ	1.8±0.6	2.3±0.6	6.0±1.3	5.0±0.6	6.9±1.3	0.6±0.3
**Th2-type**						
IL-4	2.3±0.1	35.3±8.9*	175.3±41.4	8.6±2.3*	573.4±74.1	2.7±0.2*
IL-5	2.5±1.1	2.9±0.5*	12.7±2.7	1.9±0.4*	23.6±4.2	0.8±0.1*
IL-10	1.3±0.5	2.9±0.5*	3.4±0.5	4.5±0.6	11.1±1.5	0.6±0.1*
**Pro-inflammatory**						
IL-1α	14.7±1.4	84.6±18.3*	34.0±2.3	198.9±46.6*	115.9±17.2	18.0±1.4*
IL-1β	21.0±3.8	717.5±322.7*	166.8±35.2	2056.0±492.9*	799.4±133.3	84.5±8.6*
IL-17	1.6±1.1	606.5±167.1*	16.1±3.7	1510.0±382.0*	32.4±5.1	24.1±2.5
G-CSF	11.5±1.1	302.2±54.7*	74.9±11.2	374.0±82.9*	87.9±14.7	13.4±1.4*
TNF-α	4.5±1.2	5.6±1.4	10.5±2.3	16.8±3.1	5.5±1.9	2.3±0.8
**Chemokines**						
MIP-1α	100.0±56.9	307.7±76.7*	399.6±90.6	270.6±68.4	1522.0±290.6	97.2±20.5*
MIP-1β	2.4±0.4	15.7±2.6*	9.4±1.3	13.5±2.7	14.9±1.2	1.5±0.2*
MCP-1	233.1±32.7	1509.0±247.9*	1444.0±197.2	2274.0±262.4*	2212.0±247.9	297.1±15.7*
KC	33.5±2.1	661.7±100.7*	287.5±36.0	912.1±141.2*	321.0±35.8	76.1±7.4*
RANTES	103.1±14.9	411.4±76.5*	215.8±42.6	416.0±68.6*	191.4±33.0	148.3±18.4

a Protein levels given in picograms per milliliter.

* *P*<0.05 compared to infected counterpart on same day post inoculation.

## Discussion

Studies to date have consistently suggested that Th1-type CMI is predominantly responsible for protection against pulmonary *C. neoformans* infection [6]–[10]. These studies suggested that protection against pulmonary cryptococcosis is mediated by Th1-type cytokines such as IFN-γ, TNF-α, and IL-12 [14], [16], [17], [22], [23], [50]. Additional work supported a protective role for antibody mediated immunity (AMI) against pulmonary cryptococcosis [51], [52]. The studies described herein utilize a model system in which an experimental pulmonary infection with *C. neoformans* strain H99γ in mice results in complete protection against an otherwise lethal challenge with wild-type *C. neoformans* [40], [41]. Importantly, previous studies and those presented herein show that *C. neoformans* strain H99γ is not an attenuated strain but instead induces protective host immune responses resulting in its eradication and the induction of protective anti-cryptococcal immunity. We demonstrate that all leukocyte populations appeared to be increased in protected mice, with Gr-1^+^ cells and CD11c^+^ cells comprising a significantly greater proportion of total leukocytes to respond during early infection. Recent studies by Davis and Ramakrishnan suggest that more virulent pathogens induce apoptosis of macrophages within primary granulomas resulting in the recruitment of uninfected macrophages that then phagocytose the remnants of the dead macrophages and their microbial contents before departing to seed other tissues [53]. While the “Trojan Horse” theory explaining dissemination of *C. neoformans* has been examined [54], [55], our studies show that protected mice formed well-organized granulomatous regions in which the phagocytic populations contained the infection. Pulmonary tissues derived from non-protected mice appeared to have a more dispersed leukocytic infiltrate with cryptococci replicating within the macrophages and lung alveoli. In contrast, the inflammatory response generated in protected mice subsided in correlation with the reduction of fungal burden, and no evidence of tissue destruction was observed. The zebrafish model used by Davis and Ramakrishnan does not include adaptive immune cells that may impact effector cell activity such as alternative or classical macrophage activation [53]. The pulmonary Th1-type cytokine environment observed in protected mice herein would putatively favor the induction of classically activated macrophages which have increased fungicidal activity against *C. neoformans* [56].

We evaluated the absolute role of T and B cells in the generation of protection against pulmonary *C. neoformans* infection, and found that B-cell deficient mice were able to resolve the acute infection with *C. neoformans* strain H99γ and were protected against a second pulmonary challenge with wild-type cryptococci. In contrast, T-cell deficient mice succumbed to pulmonary infection with *C. neoformans* strain H99γ, supporting a definitive role for T cells in mediating protection against acute infection. Furthermore, the inability of IL-12p40, IL-12p35, and IFN-γ deficient mice to survive pulmonary infection with *C. neoformans* strain H99γ, in contrast to the survival of IL-4R deficient mice, suggests that protective anti-cryptococcal immune responses are promoted by the induction of Th1-type cytokines. The pathogenesis of *C. neoformans* strain H99γ in immune deficient mice is comparable to that observed in mice given a similar pulmonary inocula of wild-type *C. neoformans* (unpublished observations). Also, previous studies demonstrated no differences in several phenotypes associated with cryptococcal pathogenesis in *C. neoformans* strain H99γ compared to wild-type *C. neoformans* [40] further suggesting that clearance of the acute infection with the transgenic strain was not due to attenuation. Nevertheless, the protective anamnestic immune responses were generated against the virulent *C. neoformans* strain H99 and appeared to be T cell-mediated. However, simply because B cells are not absolutely required for protection in our model does not entirely exclude any role for AMI participation in protective anti-cryptococcal immune responses. T cells more likely induce protective antibody responses that, in turn, assist to shorten the duration of the infection [51], [57]. In fact, we have previously demonstrated that *C. neoformans*-specific antibodies generated in protected mice were predominantly of a protective phenotype [41]. These antibodies could potentially limit *C. neoformans* infection in patients with reduced CMI responses if present in the correct isotype and proportions.

Nevertheless, we elected to concentrate on evaluating protective anamnestic T cell-mediated immune responses to pulmonary *C. neoformans* infection. Our results indicated that protection against a second pulmonary *C. neoformans* challenge in mice is associated with increased recruitment of APCs and leukocytes consisting of granulocytes, macrophages, CD4^+^ T lymphocytes, and CD8^+^ T lymphocytes into the lungs at days 3 and 7 post-secondary inoculation compared to non-protected mice. Current studies are underway to determine the mechanisms by which specific T cell subsets mediate protection in this model.

Upon examination of the cytokine/chemokine profile of pulmonary homogenates from protected mice, we observed a predominantly Th1/pro-inflammatory-type response. Conversely, pulmonary homogenates of non-protected mice expressed a predominantly Th-2 type cytokine profile. Th1-type and pro-inflammatory cytokines have long been associated with protection against pulmonary cryptococcosis [14], [16], [17], [22], [23], [50]. Most profound were the high levels of pro-inflammatory cytokines observed in protected mice on days 3 and 7 post-secondary inoculation compared to non-protected mice. Although we cannot assume that higher levels of pro-inflammatory cytokines directly correlates to increased biological function, it may correlate with enhanced phagocyte killing activity in protected mice. Again, the levels of Th1/pro-inflammatory cytokines subsided as the pulmonary fungal burden decreased in protected mice. Additionally, significantly increased levels of IL-17, G-CSF and KC, all known neutrophil chemoattaractants, were observed in lung homogenates from protected mice compared to non-protected mice on days 3 and 7, concurrent with increased Gr-1^+^ cells infiltrating into the lungs. Our results contrast with previous studies suggesting that increased neutrophilia during early infection results in greater inflammation and exacerbation of pulmonary cryptococcosis [58]. Further studies will determine the significance of this infiltration and the putative mechanism of Gr-1^+^ effector cell function in protected mice.

Interestingly, we did not observe significant increases in either IFN-γ or TNF-α, cytokines previously shown to be important for protection against acute pulmonary *C. neoformans* infections [14], [16], [17], [59]. However, we should note that in the previous studies, these cytokines were increased in response to primary infections with different strains of *C. neoformans* – YC-11 (a serotype A strain) [16], [17] and 52D (a serotype D strain) [14], [59]. These strains are not typically as pathogenic as *C. neoformans* strain H99 (used herein) suggesting that strain-specific cytokine responses occur. Interestingly, the lack of protection against pulmonary *C. neoformans* strain H99γ infection in IFN-γ deficient mice suggests that IFN-γ production by the transgenic strain alone is insufficient to promote protection. The IFN-γ secreted by the transgenic strain perhaps induces a cascade of events resulting in a Th1-type polarized response and protection in immune competent mice. However, IFN-γ deficient mice have other immune abnormalities (i.e., reduced macrophage activity and production of reactive oxygen species) that mitigate forming this conclusion. IFN-γ and TNF-α have been shown to be important mediators of protection against other pulmonary fungal infections. Specifically, TNF-α appears to be the chief mediator of protection in both primary and secondary pulmonary *Histoplasma capsulatum* infection in mice [60]–[64]. Vaccine induced immunity against pulmonary *Blastomyces dermatididis* infection in immune-competent animals appears to be mediated by TNF-α and IFN-γ [65]. However, protection still occurs in immune-deficient mice, suggesting that alternate and/or compensatory mechanisms of protection are present in immune-deficient hosts. We have previously observed significant increases in IFN-γ and TNF-α during primary pulmonary infection of mice with *C. neoformans* strain H99γ [40] suggesting that these cytokines may be of greater importance in protection against acute infections. Thus, while our results may not completely correlate with those observed in *C. neoformans* or other pulmonary fungal infection models, our results suggest a greater role for other cytokines in the induction of protective anamnestic responses against *C. neoformans* infections.

Until now, studies have been unable to demonstrate complete clearance of *C. neoformans* from infected tissues or 100% protection from subsequent infections with pathogenic *C. neoformans* strains. Thus, the mechanisms responsible for mediating protective anamnestic immune responses have largely been extrapolated using model systems that provide varying degrees of protection against pulmonary cryptococcosis. Our studies show that protective anti-cryptococcal immune responses are predominantly dependent on the stimulation of an early and sustained Th1-type CMI response that decreases as the infection is brought under control thereby limiting lung damage. However, the induction of protection via various pro-inflammatory cytokines and chemokines and those effector cells responsible for mediating this protection still require further study and clarification. Nonetheless, these studies clearly demonstrate the advantage of using a pathogenic fungus engineered to secrete host cytokines in order to achieve a biological “print out” of those factors that mediate protective host immunity. Such information will prove critical for the design of immune-based therapies to combat human mycoses.

## Materials and Methods

### Mice

Female BALB/c (H-2^d^) (National Cancer Institute/Charles River Laboratories and The Jackson Laboratory), CD19^−/−^ (B cell deficient), Foxn1^nu^ (T cell deficient), IL12a^tm1Jm^/J (IL-12p35 deficient), IL12b^tm1Jm^/J (IL-12p40 deficient) (The Jackson Laboratory), and IFNg^tm1Ts^/J (IFN-γ deficient; a kind gift of Dr. Bernard Arulanandam, The University of Texas at San Antonio, San Antonio, TX) mice, all on the BALB/c background with an average weight of 20–25 grams, were used throughout these studies. Mice were housed at The University of Texas at San Antonio Small Animal Laboratory Vivarium and handled according to guidelines approved by The University of Texas at San Antonio Institutional Animal Care and Use Committee.

### Strains and media

*C. neoformans* strains H99 (serotype A, Mat α) and H99γ (an interferon-gamma producing *C. neoformans* strain derived from H99 [40]) were recovered from 15% glycerol stocks stored at –80°C prior to use in the experiments described herein. The strains were maintained on yeast-extract-peptone-dextrose (YPD) media (1% yeast extract, 2% peptone, 2% dextrose, and 2% Bacto agar). Yeast cells were grown for 18–20 h at 30°C with shaking in YPD broth (Becton Dickinson and Company, Sparks, MD), collected by centrifugation, washed three times with sterile phosphate-buffered saline (PBS), and viable yeast quantified using trypan blue dye exclusion in a hemacytometer.

### Pulmonary infections

Pulmonary *C. neoformans* infections were initiated by nasal inhalation as previously described [66], [67]. Briefly, anesthetized BALB/c mice received a yeast inocula of 1×10^4^ colony-forming units (CFU) of either *C. neoformans* strain H99γ or heat-killed *C. neoformans* strain H99 (HK*C.n.*) yeasts in 50 µl of sterile PBS and were allowed 100 days to resolve the infection. Subsequently, the immunized mice received a second experimental pulmonary inoculation with 1×10^4^ CFU of wild-type *C. neoformans* strain H99 in 50 µl of sterile PBS. The inocula used for immunizations and challenge were verified by quantitative culture on YPD agar. The mice were fed ad libitum and were monitored by inspection twice daily. Mice were euthanized on days 3, 7 or 14 post-secondary inoculation, and lung tissues were excised using aseptic technique. Tissues were homogenized in 1 ml of sterile PBS, followed by culture of 10-fold dilutions of each tissue on YPD agar supplemented with chloramphenicol (Mediatech, Inc., Herndon, VA). CFU were enumerated following incubation at 30°C for 48 h. Alternatively, mice intended for survival analysis were monitored by inspection twice daily and euthanized if they appeared to be in pain or moribund. Mice were euthanized using CO_2_ inhalation.

### Pulmonary leukocyte isolation

Lungs were excised on days 3, 7, and 14 post inoculation and digested enzymatically at 37°C for 30 minutes in 10 ml of digestion buffer (RPMI 1640 and 1 mg/ml of collagenase type IV [Sigma Chemical Co., St. Louis, MO.]) with intermittent (every 10 min) stomacher homogenizations. The enzymatically-digested tissues were then successively filtered through sterile nylon filters of various pore sizes (70 and 40 µm) (BD Biosciences) and washed with sterile HBSS to enrich for leukocytes. Erythrocytes were lysed by incubation in NH_4_Cl buffer (0.859% NH_4_Cl, 0.1% KHCO_3_, 0.0372% Na_2_EDTA [pH 7.4]; Sigma) for 3 minutes on ice followed by the addition of a 10-fold excess of PBS. The resulting leukocyte population was then collected by centrifugation (800X*g*) for 5 minutes, washed twice with sterile PBS, resuspended in sterile PBS+ 2% heat-inactivated fetal bovine serum (FACS buffer) and enumerated in a hemacytometer using trypan blue dye exclusion. Flow cytometric analysis was used to determine the percentage of each leukocyte population as well as the absolute number of total leukocytes (CD45^+^) within the lung cell suspension for standardization of hemacytometer counts.

### Antibodies

For flow cytometry experiments, cells were incubated with CD16/CD32 (Fc Block™) (BD Pharmingen Corp., San Diego, CA) and the following antibodies conjugated to phycoerythrin (PE), allophycocyanin (APC), or PECy7 were added: a cocktail of CD3, CD4, and CD8α; CD45, MHC class II, Gr-1, and CD11b, (BD Pharmingen Corp.), CD11c (eBioscience Inc., San Diego, CA), and F4/80 (Caltag Laoratories, Burlingame, CA). For immunohistochemistry experiments, the following antibodies were used: CD4 rat anti-mouse (R&D Systems) and CD8 rat anti-mouse (BD Pharmingen). Primary antibodies were detected using FITC conjugated goat anti-rat IgG secondary antibody (Jackson ImunoResearch laboratories, Inc., West Grove, PA).

### Flow cytometry

Standard methodology was employed for the direct immunofluorescence of pulmonary leukocytes. Briefly, in 96-well U-bottom plates, 100 µl containing1×10^6^ leukocyte-enriched lung cells in FACS buffer were incubated with 50 µl of Fc Block™ (BD Pharmingen) diluted in FACS buffer for 5 minutes to block non-specific binding of antibodies to cellular Fc receptors. Subsequently, an optimal concentration of fluorochrome-conjugated antibodies (between 0.06–0.5 µg/1×10^6^ cells in 50 µl of FACS buffer) were added in various combinations to allow for dual or triple staining experiments and plates were incubated for 30 minutes on ice. Following incubation, the cells were washed three times with FACS buffer and cells were fixed in 200 µl of 2% ultrapure formaldehyde (Polysciences, Inc., Warrington, PA). Cells incubated with either FACS buffer alone or single fluorochrome-conjugated antibodies were used to determine positive staining and spillover/compensation calculations, and the flow cytometer determined background fluorescence. The samples were analyzed using BD FACSArray software™ on a BD FACSArray flow cytometer (BD Pharmingen). Dead cells were excluded on the basis of forward angle and 90° light scatter. For data analyses, 30,000 events (cells) were evaluated from a predominantly leukocytic population identified by backgating from CD45^+^-stained cells. The absolute number of total leukocytes was quantified by multiplying the total number of cells observed by hemacytometer counting by the percentage of CD45^+^ cells determined by flow cytometry. The absolute number of each leukocyte subset (Gr-1^+^, F4/80^+^, CD11c^+^, MHC class II^+^, CD4^+^/CD3^+^ and CD8^+^/CD3^+^ lymphocytes) was determined by multiplying the percentage of each gated population by the total number of CD45^+^ cells.

### Immunohistochemistry

Mice were euthanized at days 3, 7, and 14 post-secondary inoculation in order to excise the lungs. The pericardium and trachea were exposed by dissection and an incision was made in the trachea for the insertion of a sterile flexible cannula attached to a 3 ml syringe to slowly inflate the lungs with 0.5 to 0.7 ml of Tissue-Tek optimal tissue cutting (OCT) compound (Sakura Finetek, Torrance, CA) plus 2 M sucrose solution (1∶1, vol/vol). The lungs were then excised and immediately placed in cryomolds containing OCT medium on dry ice and then stored at −80°C until use.

Serial frozen tissue sections were cut to a thickness of 10 µm and fixed at −20°C in acetone for 10 minutes. Tissue sections were then stained using hematoxylin and eosin (The University of Texas Health Sciences Center at San Antonio Histology & Immunohistochemistry Laboratory, San Antonio, TX) or further processed for immunofluorescence analysis. Sections stained with hematoxylin and eosin were examined under a light microscope (Eclipse E400, Nikon Co, Tokyo, JAP) at high and low power magnification and microphotographs taken using Digital Microphotography system DFX1200 with ACT-1 software (Nikon).

Sections destined for immunofluorescence analysis were immediately placed in 70% ethanol for 5 minutes and washed twice in PBS for 3 minutes each. Nonspecific binding was inhibited by blocking for 30 minutes at room temperature with species-specific serum (10% in PBS) (matched with the species of the secondary antibody). Tissue sections were incubated overnight at 4°C with primary antibodies diluted in species-specific serum (3% in PBS) at pre-optimized concentrations. Subsequently, the sections were washed seven times in TRIS-NaCl-Tween 20 (TNT) buffer solution for 3 minutes per wash followed by incubation with the secondary antibodies for 30 minutes at room temperature. Slides were then washed seven times in TNT buffer for 3 minutes per wash, once in PBS containing 1% Triton X to minimize background fluorescence (3 minutes) and a final wash in TNT buffer (3 minutes). Sections were then mounted with Fluorsave reagent (Calbiochem, La Jolla, CA) containing 0.3 µM 4′,6′-diamidino-2-phenylindole dilactate (DAPI) (Molecular Probes, Eugene, OR). Fluorescence was visualized with a Leica DMR epifluorescence microscope (Leica Microsystems, Wetzlar, Germany). Images were acquired using a cooled SPOT RT charge-coupled device camera (Diagnostic Instruments Inc., Sterling Heights, MI), and they were processed and analyzed using Adobe Photoshop 7.0 (Adobe, Mountain View, CA).

### Cytokine analysis

Cytokine levels in lung tissues were analyzed using the Bio-Plex Protein Array System (Luminex-based technology) (Bio-Rad Laboratories, Hercules, CA). Briefly, lung tissue was excised and homogenized in ice-cold sterile PBS (1 ml). An aliquot (50 µl) was taken to quantify the pulmonary fungal burden and an anti-protease buffer solution (1 ml) containing PBS, protease inhibitors (inhibiting cysteine, serine, and other metalloproteinases) and 0.05% Triton X-100 was added to the homogenate. Samples were then clarified by centrifugation (800×g) for 5 minutes. Supernatants from pulmonary homogenates were assayed using the Bio-Plex Protein Array System (Bio-Rad Laboratories) for the presence of interferon (IFN)-γ, interleukin (IL)-1α, IL-1β, IL-2, IL-4, IL-5, IL-10, IL-12 p70, IL-17, tumor necrosis factor (TNF)-α, and granulocyte-colony stimulating factor [G-CSF] expression as well as chemokines (macrophage inflammatory protein [MIP]-1α, MIP-1β, macrophage chemoattractant protein [MCP]-1, keratinocyte-derived chemokine (KC), and regulated upon activation, normal T cell expressed and secreted [RANTES]).

### Statistical analysis

The unpaired Student's *t* test (two-tailed) was used to analyze fungal burden, pulmonary cell populations, and cytokine/chemokine data using GraphPad Prism version 5.00 for Windows (GraphPad Software, San Diego California USA). Survival data was analyzed using the log-rank test (GraphPad Software). Significant differences were defined as *P*<0.05.
